# PD-1 inhibitors plus oxaliplatin or cisplatin-based chemotherapy in first-line treatments for advanced gastric cancer: A network meta-analysis

**DOI:** 10.3389/fimmu.2022.905651

**Published:** 2022-08-08

**Authors:** Xiaoyu Guo, Bowen Yang, Lingzi He, Yiting Sun, Yujia Song, Xiujuan Qu

**Affiliations:** ^1^ Department of Medical Oncology, the First Hospital of China Medical University, Shenyang, China; ^2^ Key Laboratory of Anticancer Drugs and Biotherapy of Liaoning Province, the First Hospital of China Medical University, Shenyang, China; ^3^ Liaoning Province Clinical Research Center for Cancer, the First Hospital of China Medical University, Shenyang, China; ^4^ Key Laboratory of Precision Diagnosis and Treatment of Gastrointestinal Tumors, Ministry of Education, the First Hospital of China Medical University, Shenyang, China; ^5^ Clinical Cancer Research Center of Shenyang, the First Hospital of China Medical University, Shenyang, China

**Keywords:** gastric cancer, first-line, PD-1 inhibitor, oxaliplatin, cisplatin, network meta-analysis

## Abstract

**Background:**

Currently, there has been no direct comparison between programmed cell death protein 1 (PD-1) inhibitors plus different chemotherapy regimens in first-line treatments for advanced gastric cancer (AGC). This study performed a network meta-analysis (NMA) to evaluate the efficacy and safety of PD-1 inhibitors plus oxaliplatin- or cisplatin-based chemotherapy.

**Methods:**

PubMed, Embase, and the Cochrane Central Register were used to seek a series of phase III randomized controlled trials (RCTs) studying on first-line PD-1 inhibitors plus chemotherapy and phase III RCTs comparing first-line oxaliplatin and cisplatin-based chemotherapy for AGC to perform NMA. The main outcome was overall survival (OS) and other outcomes included progression-free survival (PFS), objective response rate (ORR), and treatment-related adverse events (TRAEs).

**Results:**

Eight eligible RCTs involving 5723 patients were included. Compared with PD-1 inhibitors plus cisplatin-based chemotherapy, PD-1 inhibitors plus oxaliplatin-based chemotherapy could prolong the OS without statistical significance (hazard ratio [HR]: 0.82, 95% credible interval [CI]: 0.63-1.06). However, for patients with combined positive score (CPS) ≥ 1, PD-1 inhibitors plus oxaliplatin-based chemotherapy significantly prolonged the OS (HR: 0.75, 95% CI: 0.57-0.99). PFS in PD-1 inhibitors plus oxaliplatin-based chemotherapy was significantly longer than that in PD-1 inhibitors plus cisplatin-based chemotherapy (HR: 0.72, 95% CI: 0.53-0.99). Regarding safety, the incidence of ≥ 3 TRAEs was similar between PD-1 inhibitors plus oxaliplatin-based chemotherapy and PD-1 inhibitors plus cisplatin-based chemotherapy (RR: 0.86, 95% CI: 0.66-1.12). The surface under the cumulative ranking area curve (SUCRA) indicated that PD-1 inhibitors plus oxaliplatin-based chemotherapy ranked first for OS (97.7%), PFS (99.3%), and ORR (89.0%). For oxaliplatin-based regimens, there was no significant difference between nivolumab plus oxaliplatin-based chemotherapy and sintilimab plus oxaliplatin-based chemotherapy in terms of OS, PFS, ORR, and ≥3 TRAEs.

**Conclusion:**

Compared with PD-1 inhibitors plus cisplatin-based chemotherapy, PD-1 inhibitors plus oxaliplatin-based chemotherapy significantly prolonged PFS. Considering both efficacy and safety, PD-1 inhibitors plus oxaliplatin-based chemotherapy might be a better option in the first-line treatment for AGC.

## Introduction

Patients with advanced gastric cancer (AGC) have limited treatment options and poor prognosis (1). Chemotherapy is the standard first-line treatment for AGC, with a median overall survival (OS) of less than 1 year (2). The success in application of immune checkpoint inhibitors (ICIs) in many cancer types has prompted us to explore the utility of ICIs in AGC (3, 4). The CheckMate 649 study firstly showed that programmed cell death protein 1 (PD-1) inhibitors plus chemotherapy significantly prolonged OS compared with chemotherapy alone in the first-line treatment for AGC (5). In the recent, the data of ORIENT-16 study also support the advantage of PD-1 inhibitors plus chemotherapy in the first-line treatment for AGC (6). Based on these studies, PD-1 inhibitors in combination with chemotherapy has been recommended as the first-line treatment for AGC (7).

However, not all studies of PD-1 inhibitors based first-line treatment of AGC met their primary endpoints. In the KEYNOTE-062 study, the addition of PD-1 inhibitors to chemotherapy did not significantly prolong OS (8). One of the major differences between the KEYNOTE-062 study and the CheckMate 649 or ORIENT-16 study is the platinum used in chemotherapy. Oxaliplatin-based chemotherapy was used in the CheckMate 649 and ORIENT-16 studies, whereas cisplatin-based chemotherapy was used in the KEYNOTE-062 study. Previous studies of chemotherapy alone in the first-line treatment for AGC have shown that, compared with cisplatin, oxaliplatin has more clinical benefits and considerable advantages in safety (9–11). However, it remains unknown whether oxaliplatin also has advantage when used in combination with PD-1 inhibitors.

Although PD-1 inhibitors plus chemotherapy has been recommended as the first-line treatment for AGC, the therapeutic effect still has potential for improvement by optimizing combination regimens. Prospective clinical studies should be designed to explore combined options, but such studies require a long time to obtain results. Network meta-analysis (NMA) using data analysis from published studies can quickly answer this question and provide clinical reference. Therefore, we conducted the NMA to compare the efficacy and safety of PD-1 inhibitors combined with oxaliplatin or cisplatin-based chemotherapy in the first-line immunotherapy for AGC, hoping to provide some insights for clinical treatment decisions.

## Methods

### Search strategy

This NMA was conducted following the Preferred Reporting Items for Systematic Review and Meta-Analysis (PRISMA) (12) and the PRISMA extension statement for network meta-analysis (**Supplementary Table 1**) (13). From the PubMed, Embase, and Cochrane Central Register of randomized controlled trials, we identified qualified phase III randomized controlled trials (RCTs) containing first-line PD-1 inhibitors plus chemotherapy and phase III RCTs comparing first-line oxaliplatin and cisplatin-based chemotherapy for AGC. We searched for studies using keywords including PD-1 inhibitors, oxaliplatin, cisplatin, gastric cancer, first-line and randomized controlled trial (**Supplementary Table 2**). We also searched abstracts from major conferences of the American Society of Clinical Oncology (ASCO), the European Society of Medical Oncology (ESMO), the American Association for Cancer Research (AACR), and the World Congress on Gastrointestinal Cancer (WCGC). These clinical studies were limited to those published in English before February 28, 2022.

### Inclusion and exclusion criteria

We included phase III RCTs containing first-line PD-1 inhibitors plus chemotherapy and phase III RCTs comparing first-line oxaliplatin- and cisplatin-based chemotherapy for AGC to perform NMA. These trials met the following inclusion criteria: 1) Histologically confirmed AGC. 2) Two or more different-arm studies that included PD-1inhibitors plus chemotherapy and studies comparing oxaliplatin- and cisplatin-based chemotherapy in first-line treatments. 3) The hazard ratio (HR) or relative risk (RR) and its 95% credible interval (CI) of OS, progression-free survival (PFS), objective response rate (ORR) and adverse events (AEs) were available. 4) Published articles were reported in English. Exclusion criteria: 1) Trials involving the results of radiotherapy. 2) Trials only include results from special patient populations, such as elderly patients. 3) HER2 positive AGC/GEJ cancer. 4) Research for which the published data was insufficient for analysis.

### Data extraction and quality evaluation

We extracted the design of the trial, sample size, median age, combined positive score (CPS) and primary endpoints of each treatment into a spreadsheet for further analysis. For AEs, we tended to use treatment-related adverse events (TRAEs) for analysis. When TRAEs were not reported, we used common AEs instead. We evaluated the qualities of RCTs included in the present NMA using ROB2 recommended by the Cochrane Collaboration (14). We assessed the following parameters as having a low risk, some concerns, or a high risk: 1) Bias arising from the randomization process. 2) Bias due to deviations from intended interventions. 3) Bias due to missing outcome data. 4) Bias in measuring the outcome. 5) Bias in selection of the reported result. Quality evaluation was performed independently by two investigators (XYG and BWY), where in cases of conflict, a third investigator (XJQ) was consulted for the purpose of conflict resolution.

### Statistical analysis

The primary outcome of this study was OS. Secondary outcomes were PFS, ORR and TRAEs of grade 3 and higher (≥ 3 TRAEs). NMA was performed in a Bayesian framework using a Markov Chain Monte Carlo simulation technique within the GEMTC package in the R-Statistics and the J.A.G.S. program (15). Stata 14.0 was used to graphically display the results. For each outcome, 150,000 sample iterations were generated with 100,000 burn-ins and a thinning interval of 10 (16). Fixed and random effect models were considered and compared using deviance information criteria (DIC). If the DIC difference between the random model and the fixed model was less than 5, the fixed model should be selected (17). Model convergence was assessed using a Brooks-Gelman-Rubin diagnostic plot and trace plot (18). Heterogeneity was assessed between studies using the I^2^ statistic. The estimated I^2^ values under 25%, between 25% and 50%, or over 50% indicated low, moderate, or high heterogeneity respectively (19). All treatments were ranked according to the surface under the cumulative ranking area curve (SUCRA). The higher SUCRA value meant that treatment was more likely to be ranked on the top (20).

## Results

### Literature search and study characteristics

**Figure 1:**
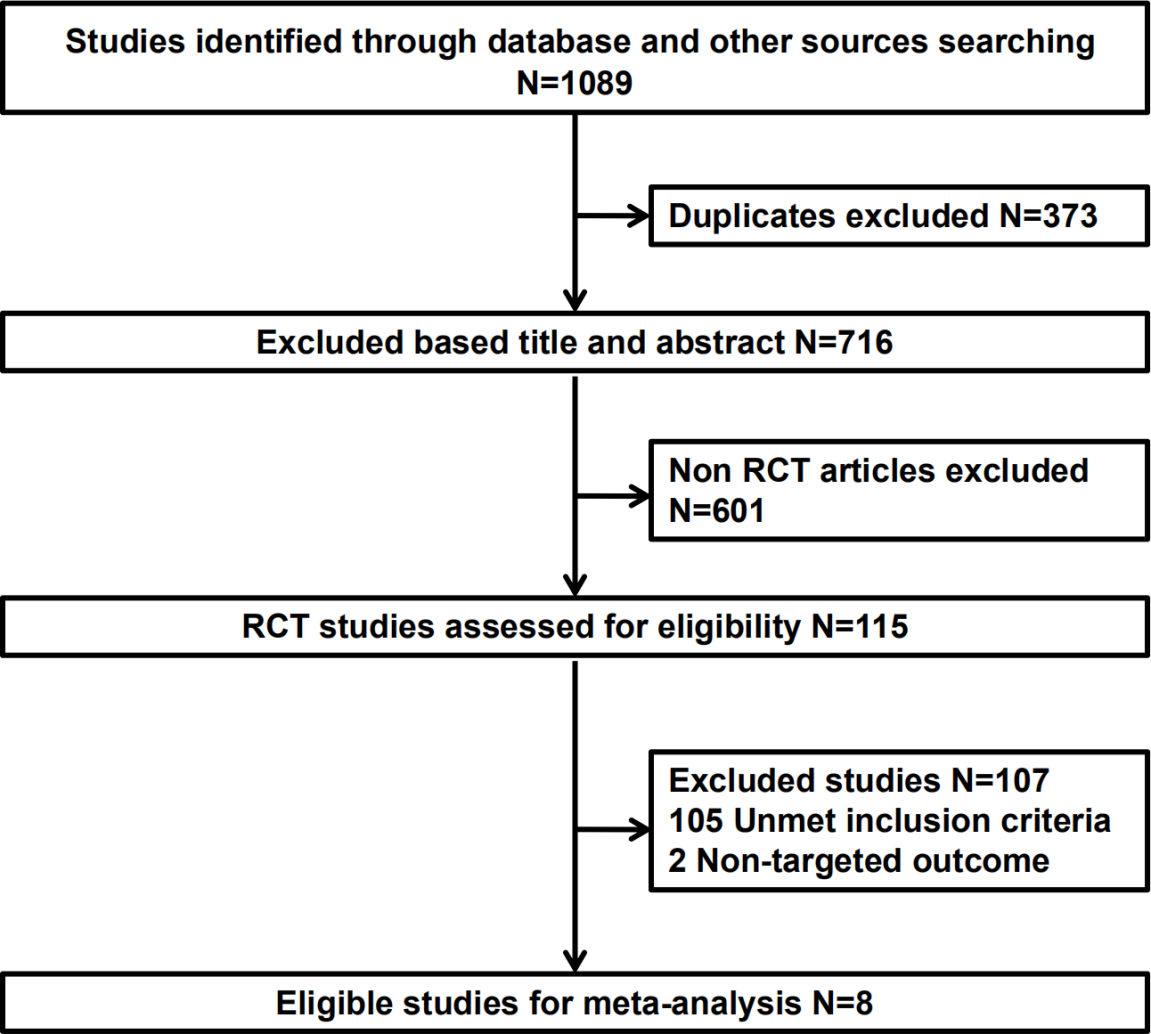
Study selection process. RCT, randomized clinical trial.

**Figure 2:**
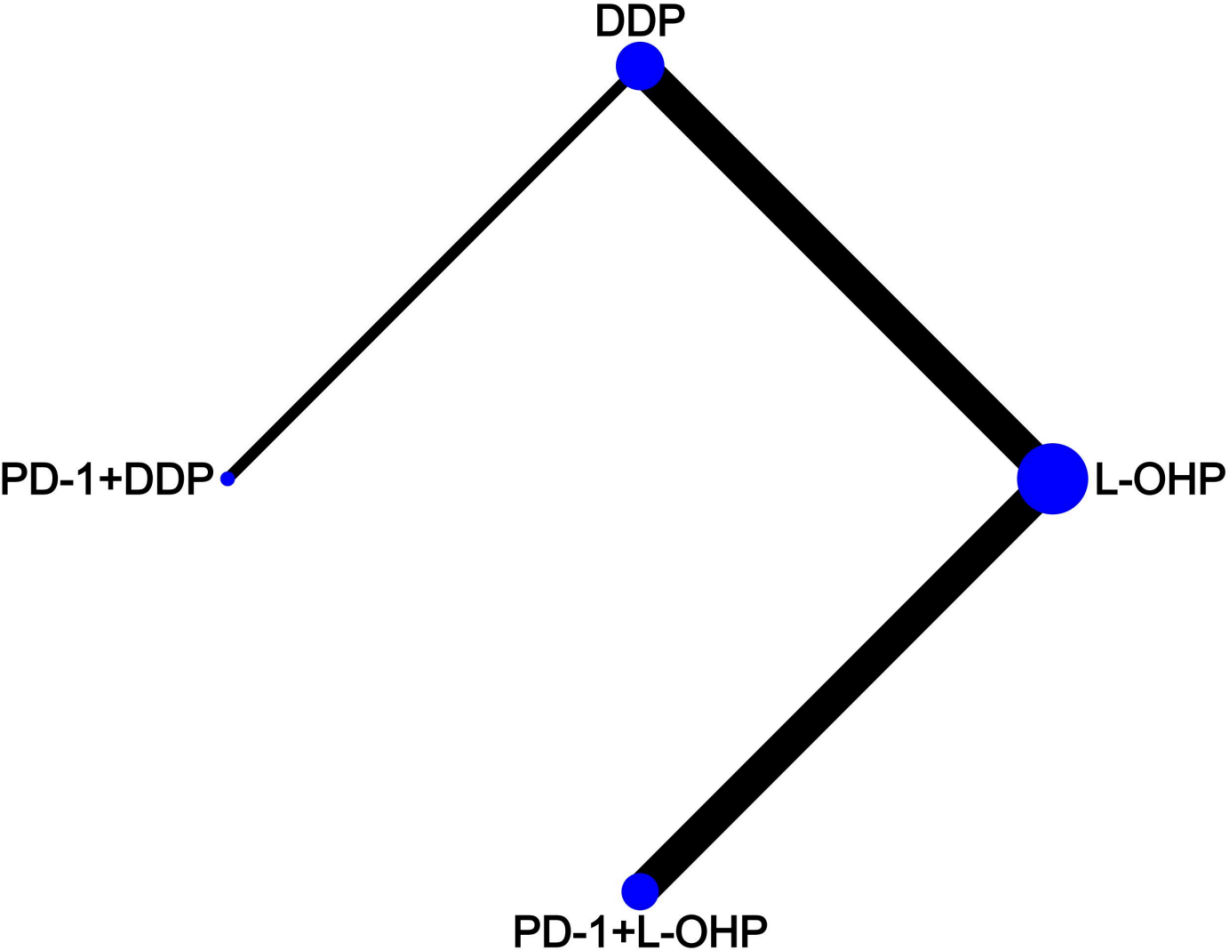
Network map. Each circular node represented a type of treatment. Circle size reflects the proportion of patients included in each treatment group. Solid lines represent randomized controlled trials (RCTs) while relative thickness represents the number of included studies. PD-1+L-OHP, PD-1 inhibitors plus oxaliplatin-based chemotherapy; PD-1+DDP, PD-1 inhibitors plus cisplatin-based chemotherapy; L-OHP, oxaliplatin-based chemotherapy; DDP, cisplatin-based chemotherapy.

Literature screening was conducted according to the PRISMA procedure (**Figure 1**). In total, eight trials involving 5723 patients met predefined inclusion criteria. Key characteristics and specific treatments for the included trials were summarized (**Table 1**). Three studies compared PD-1 inhibitors plus oxaliplatin-based chemotherapy (PD-1+L-OHP) with oxaliplatin-based chemotherapy (L-OHP), and one study compared PD-1 inhibitors plus cisplatin-based chemotherapy (PD-1+DDP) with cisplatin-based chemotherapy (DDP), four studies compared L-OHP with DDP. The four treatment regimens, including PD-1+L-OHP, PD-1+DDP, L-OHP, and DDP, formed a network map of NMA (**Figure 2**).

**Table 1:** Baseline characteristics of studies included in the systematic review.

Study	Treatment Arms	Sample size	Median age	Males No. (%)	CPS subgroup	Primary endpoints
KEYNOTE-062 (8)	Pem+PF^a^	257	62	195 (75.9)	≥1, ≥10	OS, PFS
	PF	250	62.5	179 (71.6)		
CheckMate 649 (5)	Niv+XELOX/FOLFOX^b^	789	62	540 (68)	≥1, ≥5	OS, PFS
	XELOX/FOLFOX	792	61	560 (71)		
ATTRACTION-4 (21)	Niv+CAPOX/SOX^c^	362	63.5	253 (69.9)	NA	OS, PFS
	CAPOX/SOX	362	65	270 (74.6)		
ORIENT-16 (6)	Sin+XELOX^d^	327	62	253 (77.4)	≥1, ≥5, ≥10	OS
	XELOX	323	60	230 (71.2)		
SOLAR (10)	TAS-118+oxaliplatin^e^	347	NA	251 (72)	NA	OS
	CS^f^	334	NA	218 (65)		
JapicCTI-101021 (9)	SOX^g^	343	65	240 (75.5)	NA	OS, PFS
	CS	342	65	237 (73.1)		
SOPP (22)	SOX	173	58	123 (71)	NA	PFS
	SP^h^	164	55	106 (65)		
SOX-GC (11)	SOX	279	NA	NA	NA	OS
	SP	279	NA	NA		

a Pem+PF: pembrolizumab 200 mg d1/3w+cisplatin 80 mg/m^2^ d1, fluorouracil 800 mg/m^2^/d1-5 or capecitabine 1000 mg/m^2^ d1-14/3w.

b Niv+XELOX/FOLFOX: nivolumab 360 mg/3w d1 or nivolumab 240 mg/2w d1+oxaliplatin 130 mg/m^2^ d1, capecitabine 1000 mg/m^2^ d1-14/3w or oxaliplatin 85 mg/m^2^ d1, tetrahydrofolate 400 mg/m^2^ d1, fluorouracil 1200 mg/m^2^ d1-2/2w.

c Niv+CAPOX/SOX: nivolumab 360 mg/3w d1+oxaliplatin 130 mg/m^2^ d1, capecitabine 1000 mg/m^2^ d1-14/3w or oxaliplatin 130 mg/m^2^ d1+ S-1 40 mg/m^2^ d1-14/3w.

d Sin+XELOX: sintilimab 3 mg/kg for body weight <60 kg, 200 mg for ≥60 kg d1/3w+oxaliplatin 130 mg/m^2^ d1, capecitabine 1000 mg/m^2^ d1-14/3w*6 cycles, then capecitabine 1000 mg/m^2^.

e TAS-118+oxaliplatin: TAS-118 (S-1 40–60 mg and leucovorin 25 mg) bid d1-7 + oxaliplatin 85 mg/m² d1/2 w.

f CS: S-1 40–60 mg bid d1-21+cisplatin 60 mg/m² d1 or d8/5 w.

g SOX: S-1 80–120 mg/day d1-14+oxaliplatin 130 mg/m^2^ d1/3w.

h SP: S-1 80 mg/m^2^/day d1-14+cisplatin 60 mg/m^2^ d1/3w. PFS, Progression Free Survival; OS, Overall Survival; NA, Not Available; No., number; CPS, combined positive score.

### Comparison between PD-1+L-OHP and PD-1+DDP

**Figure 3:**
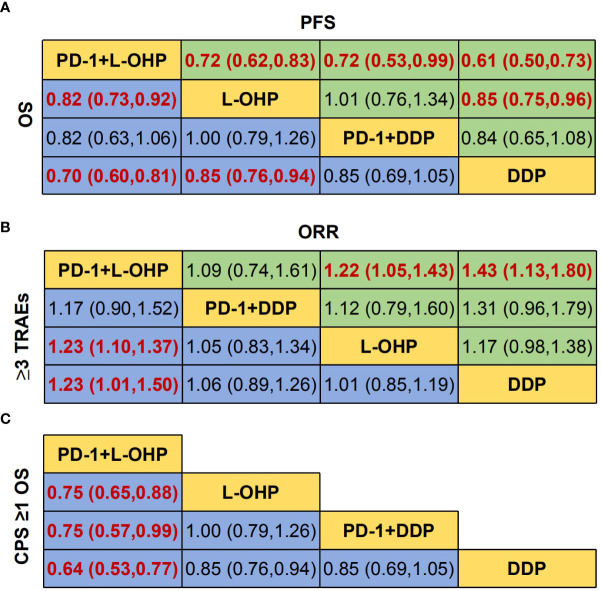
Network meta-analysis of the oxaliplatin or cisplatin-based treatments. **(A)** Hazard ratio (HR) [95% credible intervals (CI)] for overall survival (OS) and progression-free survival (PFS). **(B)** Relative risk (RR) (95% CI) for ORR and ≥3 TRAEs. **(C)** Hazard ratio (HR) [95% credible intervals (CI)] for overall survival (OS) of CPS ≥1. Data in each cell are HR or RR (95% CI) for the comparison of row-defining treatment versus column-defining treatment. HR less than 1 and RR for ORR more than 1 favored upper-row treatment. RR for ≥3 TRAEs more than 1 favored downer-row treatment. Significant results were highlighted in red and bold. PD-1+L-OHP, PD-1 inhibitors plus oxaliplatin-based chemotherapy; PD-1+DDP, PD-1 inhibitors plus cisplatin-based chemotherapy; L-OHP, oxaliplatin-based chemotherapy; DDP, cisplatin-based chemotherapy.

**Figure 4:**
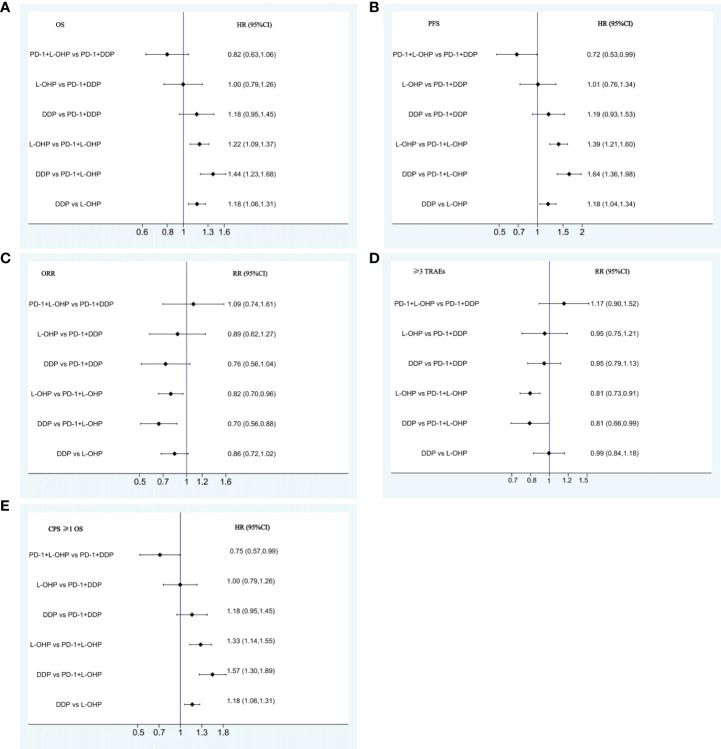
Forest plots for comparison among PD-1+L-OHP, PD-1+DDP, L-OHP, and DDP. **(A)** Forest plot for OS; **(B)** Forest plot for PFS; **(C)** Forest plot for ORR; **(D)** Forest plot for ≥3 TRAEs; **(E)** Forest plot for OS of patients with CPS ≥1. PD-1+L-OHP, PD-1 inhibitors plus oxaliplatin-based chemotherapy; L-OHP, oxaliplatin-based chemotherapy; DDP, cisplatin-based chemotherapy.

The NMA implied that compared with PD-1+DDP, PD-1+L-OHP prolonged the OS, but with no statistical significance (HR: 0.82, 95% CI: 0.63-1.06) (**Figures 3A, B**). As shown in **Figure 3C**, for patients with CPS ≥ 1, PD-1+L-OHP significantly improved the OS compared with PD-1+DDP in the first-line treatments (HR: 0.75, 95% CI: 0.57-0.99). PFS of PD-1+L-OHP was significantly longer (HR: 0.72, 95% CI: 0.53-0.99). There was no significant difference in terms of ORR between PD-1+L-OHP and PD-1+DDP (RR: 1.09, 95% CI: 0.74-1.61). As for toxicity, the incidence of ≥ 3 TRAEs was similar between PD-1+L-OHP and PD-1+DDP (RR: 1.17, 95% CI: 0.9-1.52) (**Figure 4**).

### Comparison between PD-1+L-OHP or PD-1+DDP and L-OHP/DDP

The OS of PD-1+L-OHP was significantly longer compared with L-OHP (HR: 0.82, 95% CI: 0.73-0.92) or DDP (HR: 0.70, 95% CI: 0.60-0.81). PD-1+L-OHP significantly reduced the risk of disease progression or death compared with patients treated with L-OHP (HR: 0.72, 95% CI: 0.62-0.83) or DDP (HR: 0.61, 95% CI: 0.5-0.73). The ORR of PD-1+L-OHP was significantly higher than L-OHP (RR: 1.23, 95% CI: 1.10-1.37) or DDP (RR: 1.23, 95% CI: 1.01-1.50). Compared with L-OHP (RR: 1.22, 95% CI: 1.05-1.43) or DDP (RR: 1.43, 95% CI: 1.13-1.80), PD-1+L-OHP exhibited a significantly higher incidence of ≥ 3 TRAEs (**Figures 3A, B**). However, there were no significant difference as for OS, PFS, ORR and ≥ 3 TRAEs between PD-1+DDP and L-OHP or DDP (**Figures 3A, B**, **4A–D**).

### Ranking probabilities

**Figure 5:**
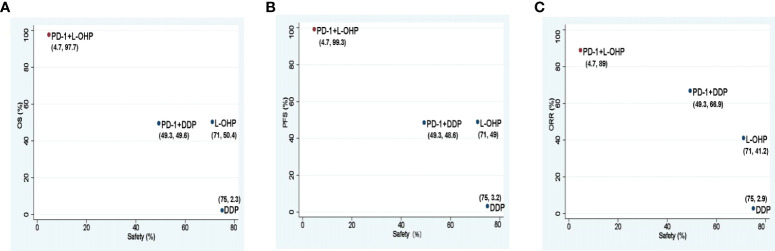
Scatter diagrams of SUCRAs among PD-1+L-OHP, PD-1+DDP, L-OHP, and DDP. **(A)** SUCRAs for safety in terms of ≥3 TRAEs and OS; **(B)** SUCRAs for safety in terms of ≥3 TRAEs and PFS; **(C)** SUCRAs for safety in terms of ≥3 TRAEs and ORR. The higher SUCRA value meant that treatment was more likely to be ranked on the top. PD-1+L-OHP, PD-1 inhibitors plus oxaliplatin-based chemotherapy; L-OHP, oxaliplatin-based chemotherapy; DDP, cisplatin-based chemotherapy; SUCRA, surface under the cumulative ranking area curve.

PD-1+L-OHP ranked first for OS (97.7%), PFS (99.3%), and ORR (89.0%). For safety, PD-1+L-OHP ranked last (4.7%) with the most incidence of ≥ 3 TRAEs (**Figure 5**).

### Comparison of different PD-1 inhibitors in combination treatments

In the current clinical studies, PD-1 inhibitors used in immune therapy plus chemotherapy regimens were also not identical, including nivolumab (Niv) in CheckMate 649 and ATTRACTION-4, pembrolizumab (Pem) in KEYNOTE-062 and sintilimab (Sin) in ORIENT-16. To ascertain whether there was a difference among combination regimens with different PD-1 inhibitors, analysis was performed for different PD-1 inhibitor combination treatments in first-line treatments for AGC (**Supplementary Figure 1**).

**Figure 6:**
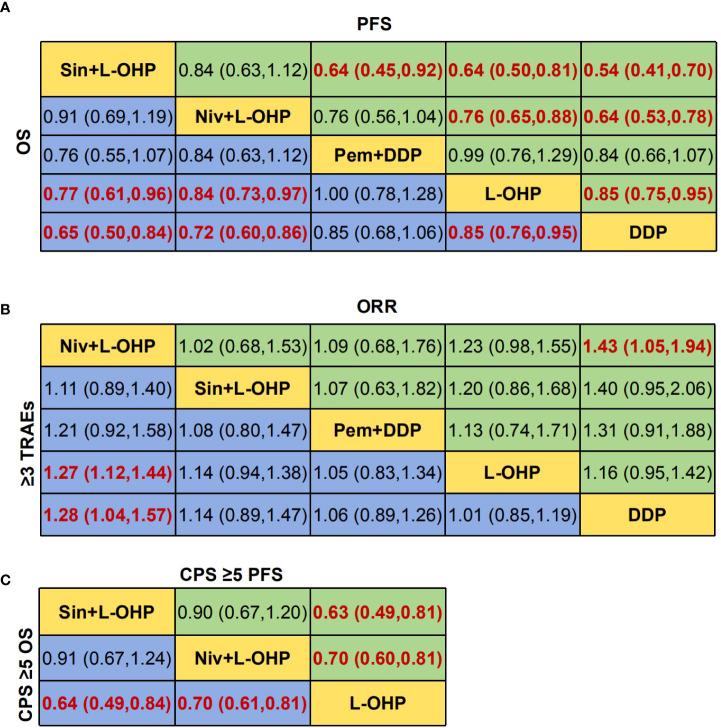
Network meta-analysis for different PD-1 inhibitor combination treatments. **(A)** Hazard ratio (HR) [95% credible intervals (CI)] for overall survival (OS) and progression-free survival (PFS). **(B)** Relative risk (RR) (95% CI) for ORR and ≥3 TRAEs. **(C)** Hazard ratio (HR) [95% credible intervals (CI)] for overall survival (OS) of CPS ≥1. Data in each cell are HR or RR (95% CI) for the comparison of row-defining treatment versus column-defining treatment. HR less than 1 and RR for ORR more than 1 favored upper-row treatment. RR for ≥3 TRAEs more than 1 favored downer-row treatment. Significant results were highlighted in red and bold. Niv+L-OHP, nivolumab plus oxaliplatin-based chemotherapy; Sin+L-OHP, sintilimab plus oxaliplatin-based chemotherapy; Pem+DDP, pembrolizumab plus cisplatin-based chemotherapy; L-OHP, oxaliplatin-based chemotherapy; DDP, cisplatin-based chemotherapy.

**Figure 7:**
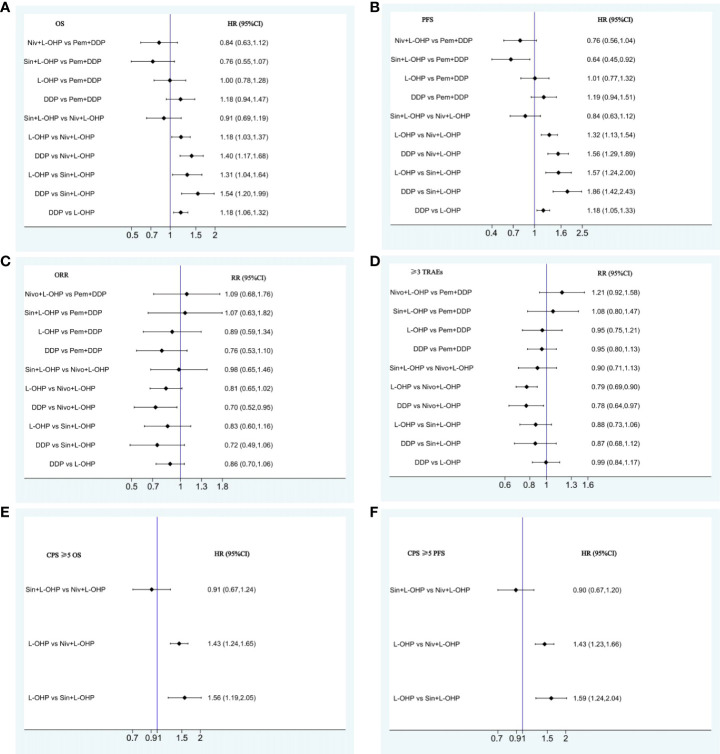
Forest plots for comparison among Niv+L-OHP, Sin+L-OHP, Pem+DDP, L-OHP, and DDP. **(A)** Forest plot for OS; **(B)** Forest plot for PFS; **(C)** Forest plot for ORR; **(D)** Forest plot for ≥3 TRAEs; **(E)** Forest plot for OS of patients with CPS ≥5; **(F)** Forest plot for PFS of patients with CPS ≥5. Niv+L-OHP, nivolumab plus oxaliplatin-based chemotherapy; Sin+L-OHP, sintilimab plus oxaliplatin-based chemotherapy; Pem+DDP, pembrolizumab plus cisplatin-based chemotherapy; L-OHP,oxaliplatin-based chemotherapy; DDP, cisplatin-based chemotherapy.

The NMA indicated that patients treated with Sin+L-OHP (HR: 0.64, 95% CI: 0.45-0.92) significantly reduced the risk of disease progression or death compared with patients treated with Pem+DDP; there was no significant difference among Niv+L-OHP, Sin+L-OHP, and Pem+DDP in terms of OS, ORR, and ≥ 3 TRAEs (**Figures 6A, B**, **7A–D**).

Patients with CPS ≥ 5 may benefit more from PD-1 inhibitors plus chemotherapy. In our analysis of patients with CPS ≥ 5, no significant difference was found between Niv+L-OHP and Sin+L-OHP in terms of OS (HR: 0.91, 95% CI: 0.67-1.24) and PFS (HR: 0.90, 95% CI: 0.67-1.20) (**Figures 6C**, **7E, F**).

**Figure 8:**
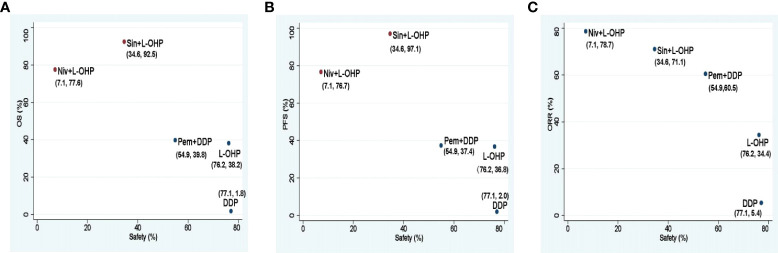
Scatter diagrams of SUCRAs among Niv+L-OHP, Sin+L-OHP, Pem+DDP, L-OHP, and DDP. **(A)** SUCRAs for safety in terms of ≥3 TRAEs and OS; **(B)** SUCRAs for safety in terms of ≥3 TRAEs and PFS; **(C)** SUCRAs for safety in terms of ≥3 TRAEs and ORR. The higher SUCRA value meant that treatment was more likely to be ranked on the top. Niv+L-OHP, nivolumab plus oxaliplatin-based chemotherapy; Sin+L-OHP, sintilimab plus oxaliplatin-based chemotherapy; Pem+DDP, pembrolizumab plus cisplatin-based chemotherapy; L-OHP,oxaliplatin-based chemotherapy; DDP, cisplatin-based chemotherapy; SUCRA, surface under the cumulative ranking area curve.

For different PD-1 inhibitors-based treatments, Sin+L-OHP ranked first for OS (92.5%), followed by Niv+L-OHP (77.6%), and Pem+DDP (39.8%). The SUCRAs for PFS indicated that Sin+L-OHP (97.1%) was the best, followed by Niv+L-OHP (76.7%), and Pem+DDP (37.4%). The SUCRAs for ORR showed that Niv+L-OHP (78.7%) was the best, followed by Sin+L-OHP (71.1%), and Pem+DDP (60.5%). For safety, Niv+L-OHP ranked the last (5.4%) with the most incidence of ≥ 3 TRAEs (**Figure 8**).

### Risk of bias assessment and sensitivity analyses

The studies included in the analysis were generally at low risk of bias (**Supplementary Figure 2**). Trace plots and Brooks-Gelman-Rubin analysis implied that the convergence of the chosen model was acceptable (**Supplementary Figure 3**). The heterogeneity of outcomes in each study was low and moderate (*I__* ^2^ < 50%).

## Discussion

PD-1 inhibitors in combination with chemotherapy has been recommended as the first-line treatment for AGC (7). However, there has been no direct comparison between PD-1 inhibitors plus different chemotherapy regimens. Therefore, we conducted the NMA to compare the efficacy and safety of PD-1 inhibitors combined with oxaliplatin or cisplatin-based chemotherapy in the first-line immunotherapy of AGC, hoping to provide some insights for clinical treatment decisions. At present, only KEYNOTE 811 has published results for first-line treatment of HER2-positive AGC, so this study did not include HER2-positive AGC for analysis (23).

The NMA suggested that compared with PD-1+DDP, PD-1+L-OHP prolonged the OS, but the result did not achieve statistical significance (HR: 0.82, 95% CI: 0.63-1.06). However, for patients with CPS ≥ 1, PD-1+L-OHP significantly prolonged the OS (HR: 0.75, 95% CI: 0.57-0.99). PFS in PD-1+L-OHP was longer than that in PD-1+L-OHP (HR: 0.72, 95% CI: 0.53-0.99). SUCRA showed that PD-1+L-OHP achieved the best OS (97.7%), PFS (99.3%) and ORR (89.0%). Regarding safety, the incidence of ≥ 3 TRAEs was similar between PD-1+L-OHP and PD-1+DDP (RR: 1.17, 95% CI: 0.9-1.52). Meanwhile, compared with L-OHP/DDP, PD-1+L-OHP achieved significant improvement in OS, PFS, and ORR, while PD-1+DDP did not significantly increase clinical benefit.

As far as we know, this is the first study comparing the efficacy and safety of PD-1+L-OHP and PD-1+DDP in first-line treatments for AGC. Our NMA showed that, compared with PD-1 inhibitors plus cisplatin-based chemotherapy, PD-1 inhibitors plus oxaliplatin-based chemotherapy has potentially higher clinical benefit. In addition, in terms of safety, the incidence of ≥ 3 TRAEs was similar between PD-1+L-OHP and PD-1+DDP, but oxaliplatin-based regimens were found to have less myelosuppression and gastrointestinal toxicity, leading to better tolerance of treatment and improved quality of life than that under cisplatin-based regimens. Considering both efficacy and safety, PD-1 inhibitors plus oxaliplatin-based chemotherapy might be a better option in the first-line treatment of AGC. This result provides a basis for clinical decision-making in the first-line treatment for AGC. However, more randomized controlled trials are needed to validate the conclusions.

Basic research has proven that oxaliplatin has a stronger ICD effect than cisplatin. Oxaliplatin can regulate the three key links of ICD through interacting with various proteins in the ICD pathway. First, after oxaliplatin enters tumor cells, it causes endoplasmic reticulum stress, and calreticulin (CRT) in the endoplasmic reticulum lumen is translocated to the cell membrane and exposed on the surface of tumor cells, triggering dendritic cells (DCs) and macrophages to engulf tumor cells. Second, oxaliplatin can induce apoptotic cells to release ATP outside the cell, the excreted ATP can recruit DCs and macrophages to the tumor site and can activate DCs. Third, in the late stage of ICD, the permeability of the cell membrane changes, and the cell nuclear high mobility group box 1 (HMGB-1) is released to the outside of the cell and is associated with DCs binding to the surface receptor TLR4, activated DCs, and significantly enhanced the proliferation of DCs. Cisplatin cannot induce CRT exposure on the cell surface, while oxaliplatin induces all three key links of ICD with a stronger ICD effect (24–26). ICD can lead to an enhanced presentation of neoantigens and activation of T cells in the tumor microenvironment (27), thereby enhancing the efficacy of ICIs. Therefore, PD-1+L-OHP may be more effective than PD-1+DDP.

Although PD-1+DDP did not provide obvious benefit in AGC, a significant OS benefit in esophageal cancer was found compared with chemotherapy alone (28). A sub-group analysis of the KEYNOTE 590 study implied that the OS of PD-1+DDP was prolonged in the esophageal adenocarcinoma sub-group, but this did not reach statistical significance, while in the esophageal squamous cell carcinoma sub-group, the OS of PD-1+DDP was significantly better than that of chemotherapy alone. Therefore, different pathological types may have different responses to the same regimen of PD-1 inhibitors plus chemotherapy. However, there is no relevant comparison of PD-1 inhibitors plus different platinum-based chemotherapy in the treatment of esophageal cancer, which warrants further exploration. In the study of esophageal cancer, the platinum-based chemotherapy regimens contained platinum plus paclitaxel and platinum plus fluorouracil. Whether platinum plus different chemotherapy drugs affected the efficiency of PD-1 inhibitor combination therapy is also worthy of exploration.

In the current clinical studies, PD-1 inhibitors used in immune therapy plus chemotherapy regimens were also not identical, including nivolumab in CheckMate 649 and ATTRACTION-4, pembrolizumab in KEYNOTE-062 and sintilimab in ORIENT-16. To improve the affinity of the antibody, the structures of different PD-1 antibodies have been modified and optimized differently. Therefore, the efficacy and safety of different PD-1 inhibitors may not be exactly the same (29). Whether there are differences among different PD-1 inhibitor combination treatments in AGC was unknown. Our analysis suggested that patients treated with Sin+L-OHP (HR: 0.64, 95% CI: 0.45-0.92) significantly reduced the risk of disease progression or death compared with patients treated with Pem+DDP; and when combined with oxaliplatin-based chemotherapy, OS, PFS, ORR and ≥ 3 TRAEs showed no significant difference between Niv+L-OHP and Sin+L-OHP within the whole population and that population with CPS ≥ 5.

Our analysis showed that Sin+L-OHP ranked first in OS and PFS, and ranked ahead of Nivo+L-OHP in safety. Both sintilimab in ORIENT-16 and nivolumab in CheckMate 649 were combined with oxaliplatin-based chemotherapy, but except for the difference of PD-1 inhibitors, the chemotherapy regimens were not identical. The chemotherapy regimen in the CheckMate 649 study was continuous double-drug chemotherapy with oxaliplatin and fluorouracil, and in the PD-1 inhibitor plus chemotherapy group, 37% of patients discontinued treatment because of AEs (5). While the chemotherapy regimen in ORIENT-16 was oxaliplatin and fluorouracil double-drug chemotherapy for six cycles, followed by fluorouracil maintenance, and only 11.6% of patients in the PD-1 inhibitor plus chemotherapy group discontinued treatment due to AEs (6). This maintenance treatment mode in ORIENT-16 had a higher treatment completion rate. Study has shown that for patients with long-term stable disease control after chemotherapy, chemotherapy can be suspended or maintenance therapy can be performed (30). And the recent ORIENT-16 study has also demonstrated the clinical benefits and feasibility of single-agent maintenance chemotherapy after double-drug chemotherapy in the first-line treatment of PD-1 inhibitors plus chemotherapy for AGC. Therefore, considering the efficacy and safety, the single-agent maintenance treatment after double-drug chemotherapy may be a more appropriate combination chemotherapy mode of first-line PD-1 inhibitors for AGC. Regarding the duration required for double-drug chemotherapy, double-drug chemotherapy was six cycles in the ORIENT-16 study. While in the CheckMate649 study, the median duration of double-drug chemotherapy in the PD-1 inhibitor plus chemotherapy group was 4-4.6 months, which was similar to the duration of double-drug chemotherapy in ORIENT-16. For elderly and frail patients, studies have shown that reducing the dose of chemotherapy drugs to 60% of the original dose did not affect the OS (31), and the reduced dose of two-drug chemotherapy is better than single-agent chemotherapy (32, 33). However, the treatment mode and dose of PD-1 inhibitors combined with chemotherapy in elderly and frail patients need to be explored in the real world, and the appropriate regimen and dose of PD-1 inhibitors combined with chemotherapy for the general population also need to be verified in future studies.

As so far, PD-1 inhibitors plus chemotherapy has not been proven to be a viable first-line treatment strategy for AGC in the general population. Thus, specific biomarkers are warranted to screen patients who will most benefit from PD-1 inhibitor combination therapy. In our analysis, PD-1+L-OHP was more beneficial for OS in the population with CPS ≥ 1. The expression levels of programmed cell death-ligand 1 (PD-L1) are the most commonly used efficacy predictive biomarkers in AGC clinical trials (34), and CPS proved to be a more useful assessment method than tumor proportion score (TPS) in determining PD-L1 expression (35). Based on sub-group analysis of the CheckMate 649 study, there was no significant benefit in the population with CPS < 5. In the JCO study of the CPS sub-group analysis of the randomized phase III trial, the benefit of the whole population was found to be mainly derived from the population with CPS ≥ 5, and the population with CPS < 5 had no significant benefit (36). Therefore, we are more inclined to recommend that patients with CPS ≥ 5 receive chemotherapy combined with PD-1 inhibitors as first-line treatment. However, the relationship between the expression of PD-L1 and the efficacy of PD-1 inhibitors in AGC is inconsistent, and the role of other predictive biomarkers warrants further evaluation (37). The values of microsatellite instability, tumor mutational burden, and mismatch repair deficiency (dMMR) as biomarkers for predicting response to PD-1 inhibitors have been confirmed by multiple studies (38, 39). The number of immune cells and the expression of T cell-related markers have been shown to be closely related to the response of immunotherapy (40–44). While the gut and tumor microbiota have also been found to be associated with immune checkpoint blockade responses (45). Therefore, the combination of multiple biomarkers may help to screen the immunotherapy advantaged population more accurately in the future.

Recent real-world studies have found that first-line PD-1 inhibitor-containing therapy may increase tumor response to the therapy of taxane plus ramucirumab, thereby improving second-line efficacy of AGC (46, 47). The use of PD-(L)1 inhibitors in front-line therapy can also improve the efficacy of subsequent chemotherapy (48), therefore, from the perspective of the overall treatment of AGC, the application of PD-1 inhibitors in first-line treatment is meaningful. Moreover, the combination of PD-1 inhibitors and chemotherapy also brings new hope to the transformation therapy of AGC. The transformation therapy of AGC refers to the transformation of unresectable gastric cancer into R0 resection by means of chemotherapy, radiotherapy, and targeted or immunotherapy, which can prolong the PFS and OS, and improve the quality of life. In pursuit of transformation, a regimen that achieves a higher ORR should be chosen. However, based on the current phase III study data, the ORR of first-line chemotherapy for AGC seems to have reached a bottleneck, and its ORR is unlikely to exceed 40%-50%. The ORR of PD-1 inhibitors combined with chemotherapy can reach 47.1%-85%, suggesting that it may become an effective transformation therapy regimen. In addition to immunotherapy combined with chemotherapy, phase II clinical studies of immunotherapy combined with different anti-angiogenic drugs have also achieved promising initial results, which deserve to be evaluated in further research (49, 50). And with the application of immunotherapy in first-line treatment for AGC, whether the continued application of ICIs can continue to benefit patients who have progressed on first-line immunotherapy also warrants further exploration. The treatment of AGC has entered a new era of immunotherapy, and we hope that personalized precision immunotherapy based on population screening and treatment optimization will bring more benefits to patients in the future.

Our study has some limitations. First, there are differences in ethnicity in the included studies, and Asians account for more in the assessed population. There are certain differences in the pathological characteristics and treatment response of gastric cancer between Eastern and Western populations, therefore our results may be more instructive for Asian populations. Second, some included studies comparing oxaliplatin and cisplatin-based chemotherapy did not provide PD-L1 expression data, so the sub-group analyses based on PD-L1 expression may be biased. The expression of PD-L1 mainly affects the efficacy of immunotherapy, and the efficacy of chemotherapy alone has not been reported to be related to PD-L1 expression. Therefore, the results based on PD-L1 expression in our analysis have certain reference significance, but further clinical studies are needed to verify this. Finally, the complete data of ORIENT-16 have not yet been published in the form of peer-reviewed articles. Thus, some of the data from this trial were extracted from the poster presentations released at the 2021 ESMO conference, and there might be some potential deviations as a result. Given these limitations, randomized controlled trials are needed to validate our results.

## Conclusions

In the first-line treatment for AGC, compared with PD-1 inhibitors plus cisplatin-based chemotherapy, PD-1 inhibitors plus oxaliplatin-based chemotherapy had no statistically significant prolongation for OS, but significantly prolonged PFS. The incidence of ≥ 3 TRAEs was similar between PD-1 inhibitors plus oxaliplatin-based chemotherapy and PD-1 inhibitors plus cisplatin-based chemotherapy. SUCRA showed that PD-1 inhibitors plus oxaliplatin-based chemotherapy achieved the best OS, PFS, and ORR. Considering both efficacy and safety, PD-1 inhibitors plus oxaliplatin-based chemotherapy might be a better option in the first-line treatment for AGC, especially for patients with CPS ≥ 1.

## Data availability statement

The original contributions presented in the study are included in the article/**Supplementary Material**. Further inquiries can be directed to the corresponding author.

## Author contributions

XQ conceived and designed the study. XG, BY, LH, YTS and YJS contributed to data acquisition, data interpretation, statistical analysis, and drafting of the manuscript. XQ contributed to data acquisition, data interpretation, statistical analysis and reviewed the manuscript. All authors contributed to the article and approved the submitted version.

## Funding

This work was supported by National Natural Science Foundation of China (81972331), "Scientist partner between China Medical University and Shenyang Branch of Chinese Academy of Sciences" Project (HZHB2022002), and Xingliao Talents Program of Liaoning Province (XLYC2008006).

## Conflict of interest

The authors declare that the research was conducted in the absence of any commercial or financial relationships that could be construed as a potential conflict of interest.

## Publisher’s Note

All claims expressed in this article are solely those of the authors and do not necessarily represent those of their affiliated organizations, or those of the publisher, the editors and the reviewers. Any product that may be evaluated in this article, or claim that may be made by its manufacturer, is not guaranteed or endorsed by the publisher.

## References

[1] Venerito M Link A Rokkas T Malfertheiner P . Review: Gastric cancer-clinical aspects. Helicobacter (2019) 24 Suppl 1:e12643. doi: 10.1111/hel.12643 31486238

[2] Smyth EC Nilsson M Grabsch HI van Grieken NC Lordick F . Gastric cancer. Lancet (2020) 396(10251):635–48. doi: 10.1016/S0140-6736(20)31288-5 32861308

[3] Ascierto PA Del Vecchio M Mandalá M Gogas H Arance AM Dalle S . Adjuvant nivolumab versus ipilimumab in resected stage IIIB-c and stage IV melanoma (CheckMate 238): 4-year results from a multicentre, double-blind, randomised, controlled, phase 3 trial. Lancet Oncol (2020) 21(11):1465–77. doi: 10.1016/S1470-2045(20)30494-0 32961119

[4] Gandhi L Rodríguez-Abreu D Gadgeel S Esteban E Felip E De Angelis F . Pembrolizumab plus chemotherapy in metastatic non-small-cell lung cancer. N Engl J Med (2018) 378(22):2078–92. doi: 10.1056/NEJMoa1801005 29658856

[5] Janjigian YY Shitara K Moehler M Garrido M Salman P Shen L . First-line nivolumab plus chemotherapy versus chemotherapy alone for advanced gastric, gastro-oesophageal unction, and oesophageal adenocarcinoma (CheckMate 649): A randomised, open-label, phase 3 trial. Lancet (2021) 398(10294):27–40. doi: 10.1016/S0140-6736(21)00797-2 34102137 PMC8436782

[6] Xu JM Jiang HP Pan YY Gu KS Cang SD Han L . Sintilimab plus chemotherapy (chemo) versus chemo as the first-line treatment for advanced gastric or gastroesophageal junction (G/GEJ) adenocarcinoma (ORIENT-16). ESMO (2021) LBA53.

[7] NCCN clinical practice guidelines in gastric cancer (Version 2.2022). Available at: http://www.nccn.org. 10.6004/jnccn.2022.0008 35130500

[8] Shitara K Van Cutsem E Bang YJ Fuchs C Wyrwicz L Lee KW . Efficacy and safety of pembrolizumab or pembrolizumab plus chemotherapy vs chemotherapy alone for patients with first-line, advanced gastric cancer: The KEYNOTE-062 phase 3 randomized clinical trial. JAMA Oncol (2020) 6(10):1571–80. doi: 10.1001/jamaoncol.2020.3370 PMC7489405 32880601

[9] Yamada Y Higuchi K Nishikawa K Gotoh M Fuse N Sugimoto N . Phase III study comparing oxaliplatin plus s-1 with cisplatin plus s-1 in chemotherapy-naïve patients with advanced gastric cancer. Ann Oncol (2015) 26(1):141–8. doi: 10.1093/annonc/mdu472 25316259

[10] Kang YK Chin K Chung HC Kadowaki S Oh SC Nakayama N . S-1 plus leucovorin and oxaliplatin versus s-1 plus cisplatin as first-line therapy in patients with advanced gastric cancer (SOLAR): A randomised, open-label, phase 3 trial. Lancet Oncol (2020) 21(7):1045–56. doi: 10.1016/S1470-2045(20)30315-6 32682457

[11] Xu RH Wang ZQ Shen L Wang W Lu JW Dai GH . S-1 plus oxaliplatin versus s-1 plus cisplatin as first-line treatment for advanced diffuse-type or mixed-type gastric/gastroesophageal junction adenocarcinoma:A randomized,phase 3 trial. J Clin Oncol (2019) 37(Suppl 15):4017. doi: 10.1200/JCO.2019.37.15_suppl.4017

[12] Moher D Liberati A Tetzlaff J Altman DG PRISMA Group . Preferred reporting items for systematic reviews and meta-analyses: The PRISMA statement. PloS Med (2009) 6(7):e1000097. doi: 10.1371/journal.pmed.1000097 19621072 PMC2707599

[13] Hutton B Salanti G Caldwell DM Chaimani A Schmid CH Cameron C . The PRISMA extension statement for reporting of systematic reviews incorporating network meta-analyses of health care interventions: Checklist and explanations. Ann Intern Med (2015) 162(11):777–84. doi: 10.7326/M14-2385 26030634

[14] Higgins JP Thomas J Chandler J Cumpston M Li T Page MJ . Cochrane handbook for systematic reviews of interventions. Hoboken, NJ, USA: John Wiley & Sons, Inc (2019). 10.1002/14651858.ED000142 PMC10284251 31643080

[15] Neupane B Richer D Bonner AJ Kibret T Beyene J . Network meta-analysis using r: A review of currently available automated packages. PloS One (2014) 9(12):e115065. doi: 10.1371/journal.pone.0115065 25541687 PMC4277278

[16] Gelman A Rubin DB . Inference from iterative simulation using multiple sequences. Stat Sci (1992) 7:457–72. doi: 10.1214/ss/1177011136

[17] Dias S Welton NJ Caldwell DM Ades AE . Checking consistency in mixed treatment comparison meta-analysis. Stat Med (2010) 29(7-8):932–44. doi: 10.1002/sim.3767 20213715

[18] Brooks S Gelman A . General methods for monitoring convergence of iterative simulations. J Comput Graphi. Stat (1998) 7:434–55. doi: 10.1080/10618600.1998.10474787

[19] Higgins JP Thompson SG Deeks JJ Altman DG . Measuring inconsistency in meta-analyses. BMJ (2003) 327(7414):557–60. doi: 10.1136/bmj.327.7414.557 PMC192859 12958120

[20] Salanti G Ades AE Ioannidis JP . Graphical methods and numerical summaries for presenting results from multiple-treatment meta-analysis: An overview and tutorial. J Clin Epidemiol (2011) 64(2):163–71. doi: 10.1016/j.jclinepi.2010.03.016 20688472

[21] Kang YK Chen LT Ryu MH Oh DY Oh SC Chung HC . Nivolumab plus chemotherapy versus placebo plus chemotherapy in patients with HER2-negative, untreated, unresectable advanced or recurrent gastric or gastro-oesophageal junction cancer (ATTRACTION-4): A randomised, multicentre, double-blind, placebo-controlled, phase 3 trial. Lancet Oncol (2022) 23(2):234–47. doi: 10.1016/S1470-2045(21)00692-6 35030335

[22] Lee KW Chung IJ Ryu MH Park YI Nam BH Oh HS . Multicenter phase III trial of s-1 and cisplatin versus s-1 and oxaliplatin combination chemotherapy for first-line treatment of advanced gastric cancer (SOPP trial). Gastric Cancer (2021) 24(1):156–67. doi: 10.1007/s10120-020-01101-4 32596783

[23] Janjigian YY Kawazoe A Yañez P Li N Lonardi S Kolesnik O . The KEYNOTE-811 trial of dual PD-1 and HER2 blockade in HER2-positive gastric cancer. Nature (2021) 600(7890):727–30. doi: 10.1038/s41586-021-04161-3 PMC8959470 34912120

[24] Tesniere A Schlemmer F Boige V Kepp O Martins I Ghiringhelli F . Immunogenic death of colon cancer cells treated with oxaliplatin. Oncogene (2010) 29(4):482–91. doi: 10.1038/onc.2009.356 19881547

[25] Gatti L Cassinelli G Zaffaroni N Lanzi C Perego P . New mechanisms for old drugs: Insights into DNA-unrelated effects of platinum compounds and drug resistance determinants. Drug Resist Update (2015) 20:1–11. doi: 10.1016/j.drup.2015.04.001 26003720

[26] Bezu L Gomes-de-Silva LC Dewitte H Breckpot K Fucikova J Spisek R . Combinatorial strategies for the induction of immunogenic cell death. Front Immunol (2015) 6:187. doi: 10.3389/fimmu.2015.00187 25964783 PMC4408862

[27] Dimeloe S Frick C Fischer M Gubser PM Razik L Bantug GR . Human regulatory T cells lack the cyclophosphamide-extruding transporter ABCB1 and are more susceptible to cyclophosphamide-induced apoptosis. Eur J Immunol (2014) 44(12):3614–20. doi: 10.1002/eji.201444879 25251877

[28] Sun JM Shen L Shah MA Enzinger P Adenis A Doi T . Pembrolizumab plus chemotherapy versus chemotherapy alone for first-line treatment of advanced oesophageal cancer (KEYNOTE-590): A randomised, placebo-controlled, phase 3 study. Lancet (2021) 398(10302):759–71. doi: 10.1016/S0140-6736(21)01234-4 34454674

[29] Chang CY Park H Malone DC Wang CY Wilson DL Yeh YM . Immune checkpoint inhibitors and immune-related adverse events in patients with advanced melanoma: A systematic review and network meta-analysis. JAMA Netw Open (2020) 3(3):e201611. doi: 10.1001/jamanetworkopen.2020.1611 32211869 PMC7097702

[30] Lu Z Zhang X Liu W Liu T Hu B Li W . A multicenter, randomized trial comparing efficacy and safety of paclitaxel/capecitabine and cisplatin/capecitabine in advanced gastric cancer. Gastric Cancer (2018) 21(5):782–91. doi: 10.1007/s10120-018-0809-y PMC6097104 29488121

[31] Hall PS Swinson D Cairns DA Waters JS Petty R Allmark C . Efficacy of reduced-intensity chemotherapy with oxaliplatin and capecitabine on quality of life and cancer control among older and frail patients with advanced gastroesophageal cancer: The GO2 phase 3 randomized clinical trial. JAMA Oncol (2021) 7(6):869–77. doi: 10.1001/jamaoncol.2021.0848 PMC8120440 33983395

[32] Hall PS Lord SR Collinson M Marshall H Jones M Lowe C . A randomised phase II trial and feasibility study of palliative chemotherapy in frail or elderly patients with advanced gastroesophageal cancer (321GO). Br J Cancer (2017) 116(4):472–8. doi: 10.1038/bjc.2016.442 PMC5318975 28095397

[33] Hwang IG Ji JH Kang JH Lee HR Lee HY Chi KC . A multi-center, open-label, randomized phase III trial of first-line chemotherapy with capecitabine monotherapy versus capecitabine plus oxaliplatin in elderly patients with advanced gastric cancer. J Geriatr Oncol (2017) 8(3):170–5. doi: 10.1016/j.jgo.2017.01.002 28119041

[34] Reck M Rodríguez-Abreu D Robinson AG Hui R Csőszi T Fülöp A . Pembrolizumab versus chemotherapy for PD-L1-Positive non-Small-Cell lung cancer. N Engl J Med (2016) 375(19):1823–33. doi: 10.1056/NEJMoa1606774 27718847

[35] Yamashita K Iwatsuki M Harada K Eto K Hiyoshi Y Ishimoto T . Prognostic impacts of the combined positive score and the tumor proportion score for programmed death ligand-1 expression by double immunohistochemical staining in patients with advanced gastric cancer. Gastric Cancer (2020) 23(1):95–104. doi: 10.1007/s10120-019-00999-9 31451991

[36] Zhao JJ Yap DWT Chan YH Tan BKJ Teo CB Syn NL . Low programmed death-ligand 1-expressing subgroup outcomes of first-line immune checkpoint inhibitors in gastric or esophageal adenocarcinoma. J Clin Oncol (2022) 40(4):392–402. doi: 10.1200/JCO.21.01862 34860570

[37] Morad G Helmink BA Sharma P Wargo JA . Hallmarks of response, resistance, and toxicity to immune checkpoint blockade. Cell (2021) 184(21):5309–37. doi: 10.1016/j.cell.2021.09.020 PMC8767569 34624224

[38] Rizvi NA Hellmann MD Snyder A Kvistborg P Makarov V Havel JJ . Cancer immunology. mutational landscape determines sensitivity to PD-1 blockade in non-small cell lung cancer. Science (2015) 348(6230):124–8. doi: 10.1126/science.aaa1348 PMC4993154 25765070

[39] Le DT Uram JN Wang H Bartlett BR Kemberling H Eyring AD . PD-1 blockade in tumors with mismatch-repair deficiency. N Engl J Med (2015) 372(26):2509–20. doi: 10.1056/NEJMoa1500596 PMC4481136 26028255

[40] Tumeh PC Harview CL Yearley JH Shintaku IP Taylor EJ Robert L . PD-1 blockade induces responses by inhibiting adaptive immune resistance. Nature (2014) 515(7528):568–71. doi: 10.1038/nature13954 PMC4246418 25428505

[41] Wen T Wang Z Li Y Li Z Che X Fan Y . A four-factor immunoscore system that predicts clinical outcome for stage II/III gastric cancer. Cancer Immunol Res (2017) 5(7):524–34. doi: 10.1158/2326-6066 28619967

[42] Chen PL Roh W Reuben A Cooper ZA Spencer CN Prieto P . Analysis of immune signatures in longitudinal tumor samples yields insight into biomarkers of response and mechanisms of resistance to immune checkpoint blockade. Cancer Discov (2016) 6(7):827–37. doi: 10.1158/2159-8290.CD-15-1545 PMC5082984 27301722

[43] Wen T Barham W Li Y Zhang H Gicobi JK Hirdler JB . NKG7 is a T-cell intrinsic therapeutic target for improving antitumor cytotoxicity and cancer immunotherapy. Cancer Immunol Res (2022) 10(2):162–81. doi: 10.1158/2326-6066 PMC8816890 34911739

[44] Zhang C Fan Y Che X Zhang M Li Z Li C . Anti-PD-1 Therapy response predicted by the combination of exosomal PD-L1 and CD28. Front Oncol (2020) 10:760. doi: 10.3389/fonc.2020.00760 32528882 PMC7266952

[45] Nejman D Livyatan I Fuks G Gavert N Zwang Y Geller LT . The human tumor microbiome is composed of tumor type-specific intracellular bacteria. Science (2020) 368(6494):973–80. doi: 10.1126/science.aay9189 PMC7757858 32467386

[46] Sasaki A Kawazoe A Eto T Okunaka M Mishima S Sawada K . Improved efficacy of taxanes and ramucirumab combination chemotherapy after exposure to anti-PD-1 therapy in advanced gastric cancer. ESMO Open (2020) 4(Suppl 2):e000775. doi: 10.1136/esmoopen-2020-000775 32719002 PMC7381840

[47] Kankeu Fonkoua LA Chakrabarti S Sonbol MB Kasi PM Starr JS Liu AJ . Outcomes on anti-VEGFR-2/paclitaxel treatment after progression on immune checkpoint inhibition in patients with metastatic gastroesophageal adenocarcinoma. Int J Cancer (2021) 149(2):378–86. doi: 10.1002/ijc.33559 PMC8488901 33739449

[48] Kato K Narita Y Mitani S Honda K Masuishi T Taniguchi H . Efficacy of cytotoxic agents after progression on anti-PD-(L)1 antibody for pre-treated metastatic gastric cancer. Anticancer Res (2020) 40(4):2247–55. doi: 10.21873/anticanres.14187 32234921

[49] Hara H Shoji H Takahari D Esaki T Machida N Nagashima K . Phase I/II study of ramucirumab plus nivolumab in patients in second-line treatment for advanced gastric adenocarcinoma (NivoRam study). J Clin Oncol (2019) 37(Suppl 4):129. doi: 10.1200/JCO.2019.37.4_suppl.129

[50] Fukuoka S Hara H Takahashi N Kojima T Kawazoe A Asayama M . Regorafenib plus nivolumab in patients with advanced gastric or colorectal cancer: An open-label, dose-escalation, and dose-expansion phase ib trial (REGONIVO, EPOC1603). J Clin Oncol (2020) 38(18):2053–61. doi: 10.1200/JCO.19.03296 32343640

