# Supplementary material for: PD-1 inhibitors plus oxaliplatin or cisplatin-based chemotherapy in first-line treatments for advanced gastric cancer: A network meta-analysis
Source: Front Immunol. 2022 Aug 8;13:905651. doi: 10.3389/fimmu.2022.905651 (PMC9393421; doi:10.3389/fimmu.2022.905651)
Supplement: Supplementary file 1 [file DataSheet_1.pdf]

Supplementary Material

Supplementary Table 1. Checklist of the PRISMA extension for network meta-analysis.

Section/topic	#	Checklist item	Reported on page #
**TITLE**			
Title	1	Identify the report as a systematic review, meta-analysis, or both.	1
**ABSTRACT**			
Structured summary	2	Provide a structured summary including, as applicable: background; objectives; data sources; study eligibility criteria, participants, and interventions; study appraisal and synthesis methods; results; limitations; conclusions and implications of key findings; systematic review registration number.	1-2
**INTRODUCTION**			
Rationale	3	Describe the rationale for the review in the context of what is already known.	2
Objectives	4	Provide an explicit statement of questions being addressed with reference to participants, interventions, comparisons, outcomes, and study design (PICOS).	2
**METHODS**			
Protocol and registration	5	Indicate if a review protocol exists, if and where it can be accessed (e.g., Web address), and, if available, provide registration information including registration number.	2-3
Eligibility criteria	6	Specify study characteristics (e.g., PICOS, length of follow-up) and report characteristics (e.g., years considered, language, publication status) used as criteria for eligibility, giving rationale.	3
Information sources	7	Describe all information sources (e.g., databases with dates of coverage, contact with study authors to identify additional studies) in the search and date last searched.	3
Search	8	Present full electronic search strategy for at least one database, including any limits used, such that it could be repeated.	3
Study selection	9	State the process for selecting studies (i.e., screening, eligibility, included in systematic review, and, if applicable, included in the meta-analysis).	3
Data collection process	10	Describe method of data extraction from reports (e.g., piloted forms, independently, in duplicate) and any processes for obtaining and confirming data from investigators.	3
Data items	11	List and define all variables for which data were sought (e.g., PICOS, funding sources) and any assumptions and simplifications made.	3
Geometry of the network	S1	Describe methods used to explore the geometry of the treatment network under study and potential biases related to it. This should include how the evidence base has been graphically summarized for presentation, and what characteristics were compiled and used to describe the evidence base to readers.	3
Risk of bias in individual studies	12	Describe methods used for assessing risk of bias of individual studies (including specification of whether this was done at the study or outcome level), and how this information is to be used in any data synthesis.	3
Summary measures	13	State the principal summary measures (e.g., risk ratio, difference in means).	3
Synthesis of results	14	Describe the methods of handling data and combining results of studies, if done, including measures of consistency (e.g., I^2) for each meta-analysis.	3
Assessment of Inconsistency	S2	Describe the statistical methods used to evaluate the agreement of direct and indirect evidence in the treatment network(s) studied. Describe efforts taken to address its presence when found.	3

Section/topic	#	Checklist item	Reported on page #
Risk of bias across studies	15	Specify any assessment of risk of bias that may affect the cumulative evidence (e.g., publication bias, selective reporting within studies).	3
Additional analyses	16	Describe methods of additional analyses (e.g., sensitivity or subgroup analyses, meta-regression), if done, indicating which were pre-specified.	3
**RESULTS**			
Study selection	17	Give numbers of studies screened, assessed for eligibility, and included in the review, with reasons for exclusions at each stage, ideally with a flow diagram.	3-4
Presentation of network structure	S3	Provide a network graph of the included studies to enable visualization of the geometry of the treatment network.	3-4
Summary of network geometry	S4	Provide a brief overview of characteristics of the treatment network. This may include commentary on the abundance of trials and randomized patients for the different interventions and pairwise comparisons in the network, gaps of evidence in the treatment network, and potential biases reflected by the network structure.	3-4
Study characteristics	18	For each study, present characteristics for which data were extracted (e.g., study size, PICOS, follow-up period) and provide the citations.	4
Risk of bias within studies	19	Present data on risk of bias of each study and, if available, any outcome level assessment (see item 12).	6
Results of individual studies	20	For all outcomes considered (benefits or harms), present, for each study: (a) simple summary data for each intervention group (b) effect estimates and confidence intervals, ideally with a forest plot.	3-6
Synthesis of results	21	Present results of each meta-analysis done, including confidence intervals and measures of consistency.	3-6
Risk of bias across studies	22	Present results of any assessment of risk of bias across studies (see Item 15).	6
Additional analysis	23	Give results of additional analyses, if done (e.g., sensitivity or subgroup analyses, meta-regression [see Item 16]).	5-6
**DISCUSSION**			
Summary of evidence	24	Summarize the main findings including the strength of evidence for each main outcome; consider their relevance to key groups (e.g., healthcare providers, users, and policy makers).	6-11
Limitations	25	Discuss limitations at study and outcome level (e.g., risk of bias), and at review-level (e.g., incomplete retrieval of identified research, reporting bias).	11
Conclusions	26	Provide a general interpretation of the results in the context of other evidence, and implications for future research.	11
**FUNDING**			
Funding	27	Describe sources of funding for the systematic review and other support (e.g., supply of data); role of funders for the systematic review.	12

Supplementary Table 2. Literature search strategies on PubMed

(((((((((("Stomach Neoplasms"[Mesh]) OR ("Gastric Neoplasms"[Title/Abstract])) OR ("Stomach Cancers"[Title/Abstract])) OR ("Gastric Cancer"[Title/Abstract])) OR ("gastroesophageal junction cancer"[Title/Abstract])) OR ("Cancer of the Stomach"[Title/Abstract])) OR ("adenocarcinoma of the stomach"[Title/Abstract])) OR ("adenocarcinoma of the gastroesophageal junction"[Title/Abstract])) OR (gastic cancer[Title/Abstract]))) AND ((((previously untreated[Title/Abstract]) OR (First-line[Title/Abstract])) OR (first-line[Title/Abstract]))))) AND (((oxaliplatin[Title/Abstract]) OR (cisplatin[Title/Abstract])))

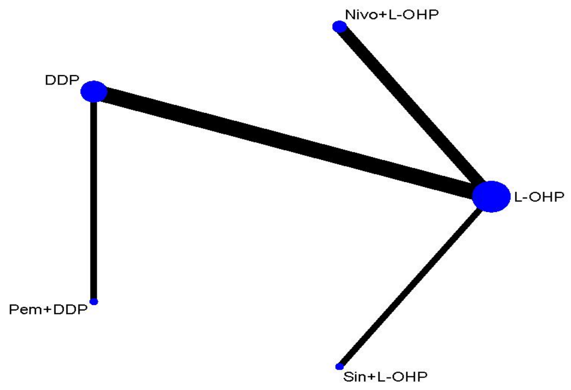

Supplementary Figure 1. Network map for different PD-1 inhibitors combined treatments. Each circular node represented a type of treatment. Circle size reflects the proportion of patients included in each treatment group. Solid lines represent randomized controlled trials (RCTs) while relative thickness represents the number of included studies. Niv+L-OHP, nivolumab plus oxaliplatin-based chemotherapy; Sin+L-OHP, sintilimab plus oxaliplatin-based chemotherapy; Pem+DDP, pembrolizumab plus cisplatin-based chemotherapy; L-OHP,oxaliplatin-based chemotherapy; DDP, cisplatin-based chemotherapy.

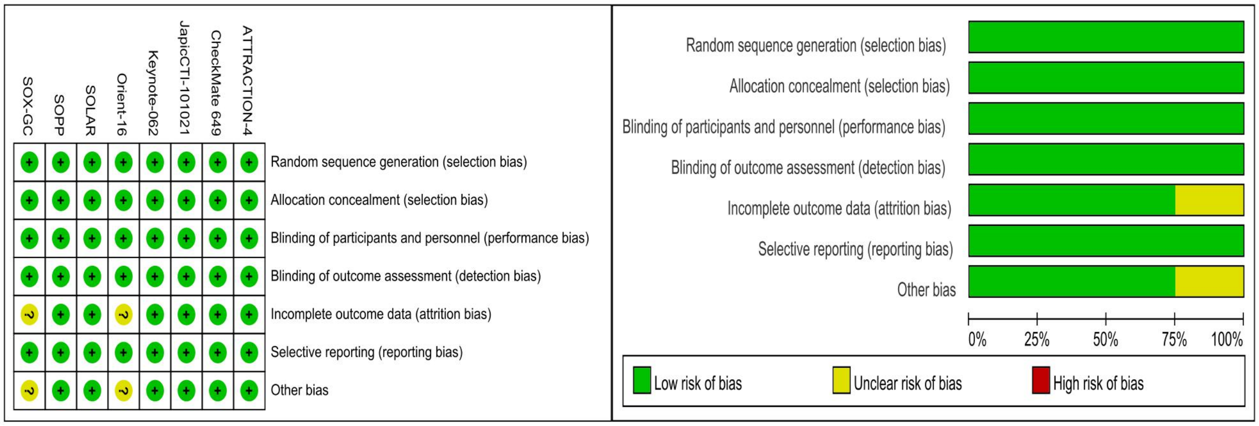

Supplementary Figure 2. Risk of bias summery. +: low risk; ?: Unclear risk; -: high risk.

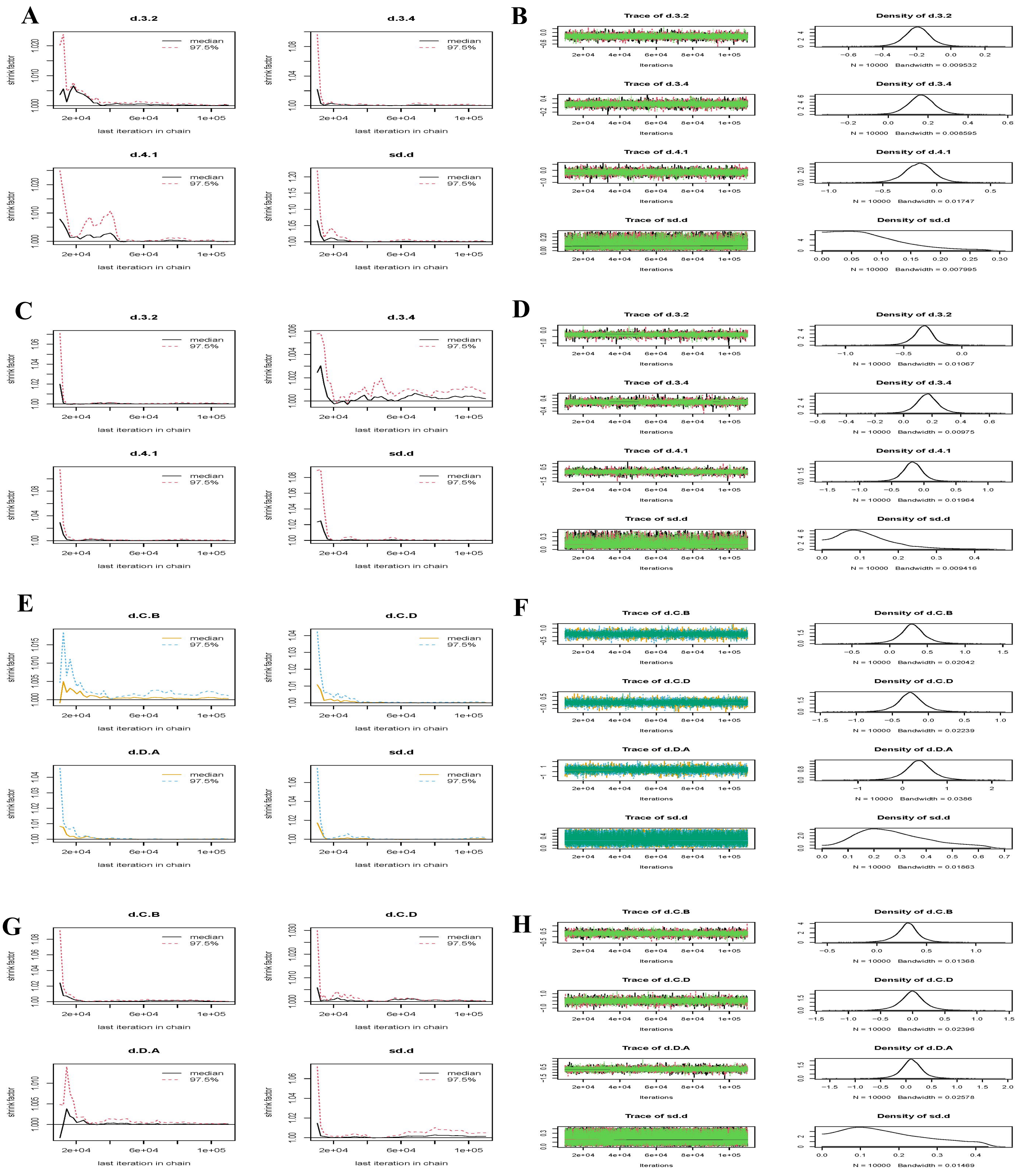

Supplementary Figure 3. Convergence of the four chains established by inspection of the Brooks-Gelman-Rubin diagnostic and the density trace plot. (A-B) OS; (C-D) PFS; (E-F) ORR; (G-H) ≥3TRAEs.
